# 晚期非小细胞肺癌抗血管生成药物治疗中国专家共识（2019版）

**DOI:** 10.3779/j.issn.1009-3419.2019.07.01

**Published:** 2019-07-20

**Authors:** 中国临床肿瘤学会血管靶向治疗专家委员会, 非小细胞肺癌专家委员会, 非小细胞肺癌抗血管生成药物治疗专家组

## 1 引言

肺癌是目前全球最常见和致死率最高的恶性肿瘤。2018年全球肺癌新发病例高达209万余例，肺癌造成的死亡人数高达176万余例^[1]^。肺癌是我国男性最常见的癌症，2018年我国约有77.4万的新增肺癌病例，约有69万人死于肺癌^[2]^。

非小细胞肺癌（non-small cell lung cancer, NSCLC）是肺癌中最常见的组织学类型，在肺癌病例中占比超过80%^[3]^。由于NSCLC的侵袭性较高，且缺乏有效的早期筛查方案，导致我国68%的肺癌患者确诊时已是晚期^[4]^。

以铂类为基础的双药化疗方案是治疗晚期NSCLC的传统标准方案，但对应的5年生存率依然很低，不足5%^[5]^。在过去几十年间，越来越多的证据显示新生血管生成在多种实体肿瘤的生长、增殖和转移中发挥着关键作用^[6]^。抗血管生成药物可作用于肿瘤微环境，使现有肿瘤血管退化，同时抑制肿瘤新生血管生成。临床数据^[6]^显示，抗血管生成药物与其他NSCLC系统治疗药物（包括化疗、小分子靶向治疗、免疫治疗）联合使用可发挥更好的抗肿瘤作用，延缓耐药，且不良反应可管理。

目前已有3个抗血管生成药物在我国获批用于治疗晚期NSCLC患者，包括血管内皮生长因子（vascular endothelial growth factor, VEGF）抑制剂贝伐珠单抗^[7]^、重组人血管内皮抑制素^[8]^和小分子多靶点酪氨酸激酶抑制剂（tyrosine kinase inhibitor, TKI）安罗替尼^[9]^。这三种药品均已进入国家医保目录。因此，随着我国抗血管生成药物的不断发展以及药物可及性的不断提高，抗血管生成药物作为晚期NSCLC患者不可或缺的治疗手段之一，有必要总结符合我国临床实际的共识内容，以指导我国肺癌治疗相关的临床科室合理使用抗血管生成药物，进一步提高我国肺癌规范化诊疗水平。为此，我们组织专家组撰写了本共识。

## 2 方法学

本共识撰写的第一步是由共识专家组组长与执笔作者基于已发表的临床研究证据，并结合临床经验，整理出共识初稿。之后由46名指南撰写专家组成员经过5轮专家组会议对共识初稿内容进行讨论和修改，最终确定共识推荐内容。因此，本共识提出的推荐内容是基于现有的中国和国际临床高质量循证医学证据以及专家组广泛认可的临床经验。

通过PubMed、EMBASE、万方医学数据库和中国知网进行文献检索，检索截止时间为2018年9月25日。在PubMed和EMBASE使用的检索式为：（‘bevacizumab’ OR‘avastin’）AND ‘non small cell lung cancer’；“（‘recombinant endostatin’ OR ‘endostar’）AND non small cell lung cancer”；“（anlotinib）AND non small cell lung cancer”。在万方医学网和中国知网使用的检索式为：“（摘要：安维汀或摘要：贝伐单抗或摘要：贝伐珠单抗）和（标题：肺癌）”；“（摘要：恩度或摘要：重组人血管内皮抑制素或摘要：重组人血管内皮抑素或摘要：重组血管内皮抑制素）和（标题：肺癌）”；“（摘要：福可维或摘要：安罗替尼）和（标题：肺癌）”。检索的文献限于系统综述、荟萃分析和随机对照临床研究，剔除重复文献、述评、编辑点评、来信、新闻报道、叙述性综述以及后续未发表于同行评审期刊的会议摘要。最后得到贝伐珠单抗、重组人血管内皮抑制素、安罗替尼相关的英文文献分别为204篇、19篇、4篇；中文文献分别为78篇、207篇、1篇。

本共识推荐内容参照“CSCO证据级别”^[10]^（表 1）。

**1:** 证据级别 Evidence level

1A类证据	基于高水平证据（严谨的*Meta*分析或RCT结果），专家组有统一共识
1B类证据	基于高水平证据（严谨的*Meta*分析或RCT结果），专家组有小争议
2A类证据	基于低水平证据，专家组有统一认识
2B类证据	基于低水平证据，专家组无统一认识，但争议不大
3类证据	专家组存在较大争议
RCT：随机对照试验。	

## 3 晚期NSCLC的抗血管生成药物的发展现状

当前NSCLC的抗血管生成治疗主要包括三大类：①靶向VEGF-血管内皮生长因子受体（vascular endothelial growth factor receptor, VEGFR）的大分子单克隆抗体；②靶点包括VEGFR的多靶点小分子TKI；③重组人血管内皮抑制素。

**靶向VEGF-VEGFR的大分子单克隆抗体**

针对VEGF-VEGFR通路的大分子单克隆抗体类药物是研究较早，也是应用较为成熟的血管靶向药物。贝伐珠单抗是首个也是唯一一个被批准应用于晚期NSCLC一线治疗的VEGF单克隆抗体。贝伐珠单抗是人源化抗VEGF的单克隆抗体，可通过中和VEGF从而达到抑制肿瘤血管生长的作用^[11]^。多项大型、前瞻性研究^[11-18]^证实，贝伐珠单抗与细胞毒类药物、TKI及免疫检查点抑制剂联合使用，可显著延长患者的无进展生存时间（progression-free survival, PFS）和/或总生存时间（overall survival, OS）。

雷莫芦单克隆抗体是另一个已获批用于局部晚期或转移性NSCLC的药物，该药主要靶向阻断VEGF与VEGFR2的结合^[19]^，从而抑制血管的生成及迁移。美国已批准该药联合多西他赛用于晚期NSCLC的二线治疗。

**靶点包括VEGFR的多靶点小分子TKI**

小分子TKI可与胞内段酪氨酸激酶域竞争性结合，抑制其磷酸化过程，阻断细胞下游信号转导通路的激活，从而抑制肿瘤血管生成^[20]^。除了与血管生成关系密切的VEGF/VEGFR以外，血小板源性生长因子/受体（platelet-derived growth factor/receptor, PDGF/PDGFR）、成纤维细胞生长因子/受体（fibroblast growth factor/receptor, FGF/FGFR）及c-Kit等均是这类药物的作用靶点^[20]^。由于不具有明显的选择性，因此这一类药物的不良反应通常较单靶点药物明显，从而限制了其在临床试验中的剂量。目前，除安罗替尼等少数药物获得阳性结果外^[21, 22]^，大部分VEGFR-TKI药物在临床研究中显示单独应用或与细胞毒类药物联合应用并不能改善晚期NSCLC患者预后。安罗替尼是国内目前唯一一个获批用于晚期NSCLC治疗的VEGFR-TKI类药物。

**重组人血管内皮抑制素**

肿瘤血管的生长过程既受到VEGF/VEGFR等因子的正向调节，也受到血管内皮抑制素等的负向调节^[23]^，因此，外源性补充抑制血管生长的细胞因子亦可以有效针对肿瘤血管生成。我国自主生产的重组人血管内皮抑制素恩度已获批应用于肺癌临床。

## 4 NSCLC抗血管生成药物临床应用推荐

本共识旨在为我国的临床医师在临床使用抗血管生成药物治疗NSCLC患者提供规范指引，因此只针对已在我国批准NSCLC治疗适应证的3个药物，即贝伐珠单抗、重组人血管内皮抑制素、安罗替尼展开，推荐意见基于我国的临床数据以及国际重要研究结果。

**一线治疗**

**贝伐珠单抗用于晚期NSCLC一线治疗的临床证据和推荐意见**

贝伐珠单抗联合含铂双药化疗方案用于晚期非鳞NSCLC患者中的疗效已在多项Ⅲ期临床研究中得到确认。Ⅲ期ECOG4599研究共纳入878例复发性或晚期非鳞NSCLC患者，数据显示，卡铂+紫杉醇联合贝伐珠单抗一线治疗比较单纯化疗方案可明显延长患者生存时间（中位OS 12.3个月*vs* 10.3个月，HR=0.79，*P*=0.003；中位PFS 6.2个月*vs* 4.5个月，HR=0.66，*P* < 0.001），提高客观缓解率（objective response rate, ORR）（35% *vs* 15%, *P* < 0.001）^[12]^。

2009年发表的一项Ⅲ期随机对照研究（AVAiL研究^[13, 14]^）进一步支持了贝伐珠单抗联合含铂双药化疗方案一线治疗在欧洲晚期非鳞NSCLC患者中的效果。该研究在1, 043例患者中开展，受试者被随机分配接受顺铂+吉西他滨化疗方案或贝伐珠单抗（两个剂量组：7.5 mg/kg、15 mg/kg）联合化疗方案。数据显示，相比单用化疗方案组（中位PFS为6.1个月），贝伐珠单抗联合化疗方案组的中位PFS明显延长（较低剂量组为6.7个月，较高剂量组为6.5个月），*P*值分别为0.003和0.03，ORR也较单用化疗组提高，7.5 mg/kg组、15 mg/kg组和单纯化疗组的ORR分别为34.1%、30.4%和20.1%。

贝伐珠单抗联合培美曲塞+卡铂/顺铂对晚期NSCLC患者的疗效在两项大型Ⅲ期研究（Pointbreak研究^[24]^和AVAPERL研究^[25]^）中得到确认。Pointbreak研究的意向治疗人群分析结果显示，贝伐珠单抗联合培美曲塞+卡铂方案一线治疗与贝伐珠单抗联合紫杉醇+卡铂方案达到疾病控制而接受后续维持治疗的患者比例相当（62% *vs* 64%）^[24]^。从AVAPERL研究的诱导治疗期（贝伐珠单抗联合培美曲塞+顺铂治疗4个周期）数据来看，这一一线治疗方案用于Ⅲb期/Ⅳ期非鳞NSCLC患者可达到较高的疾病控制率（71.9%），ORR达到22.7%^[25]^。

贝伐珠单抗联合含铂双药化疗方案在亚裔或中国的晚期非鳞NSCLC患者人群中则显示出较西方患者人群更好的疗效结果。中国的多中心随机、双盲、安慰剂对照Ⅲ期BEYOND研究^[11]^，该研究纳入276例局部晚期、转移性或复发性晚期非鳞NSCLC患者。结果显示，与单纯化疗组比较，贝伐珠单抗联合含铂双药化疗组的中位PFS明显延长（9.2个月*vs* 6.5个月，HR=0.40，*P* < 0.001），客观缓解率明显提高（54% *vs* 26%），中位OS明显延长（24.3个月*vs* 17.7个月，HR=0.68，*P*=0.0154）。AVAiL研究纳入了1, 043例患者，其中105例为亚裔患者，这一人群使用贝伐珠单抗联合含铂双药化疗方案的中位PFS结果（7.5 mg/kg组8.5个月，15 mg/kg组8.2个月）优于研究总人群结果（7.5 mg/kg组6.7个月，15 mg/kg组6.5个月）^[26]^。一项纳入了29项研究的荟萃分析^[27]^结果显示，贝伐珠单抗联合的含铂化疗方案无论是否包含紫杉醇，用于非鳞NSCLC一线治疗均可获得良好的疗效。

上述临床试验结果也得到了临床实践数据的支持。全球多中心Ⅳ期SAiL研究纳入2, 212例局部晚期、转移性和复发性非鳞NSCLC初治患者，其中有198例是中国患者。结果发现，中国患者亚组的中位OS（18.5个月）和中位至疾病进展时间（time to progression, TTP）（8.8个月）均优于研究总人群数据（中位OS和TTP分别为14.6个月和7.8个月）^[28]^。最近发表的一项中国真实世界数据研究^[29]^对149例晚期非鳞NSCLC患者的病历资料进行分析，比较一线使用含或不含贝伐珠单抗方案对临床结局的影响。结果发现，与一线不含贝伐珠单抗方案组比较，含贝伐珠单抗一线治疗将患者的中位PFS从7.0个月延长至9.7个月，组间差异显著（*P*=0.018, 4）；在野生型基因患者中，含贝伐珠单抗方案的中位PFS同样明显优于对照组（11.3个月*vs* 5.5个月，*P*=0.023, 4）。

**推荐意见1**

**在驱动基因突变阴性、PS 0分-1分的晚期NSCLC患者中，推荐贝伐珠单抗联合含铂双药方案作为一线治疗选择[1A]。**

EGFR-TKI如厄洛替尼、吉非替尼、埃克替尼、阿法替尼是*EGFR*突变阳性的晚期非鳞NSCLC患者的一线治疗药物^[30]^，然而由于后续出现的耐药现象，EGFR-TKI单药治疗对应的中位PFS仍难以突破11个月^[31]^，这也成为目前临床实践中使用EGFR-TKI的瓶颈。两项日本研究为解决这一问题提供了方向。Ⅱ期JO25567研究^[32, 33]^纳入152例*EGFR*敏感型突变（19外显子缺失或L858R突变阳性）的Ⅲb期/Ⅳ期非鳞NSCLC患者，比较厄洛替尼联合贝伐珠单抗或单用厄洛替尼的疗效及安全性。2018年美国临床肿瘤学会（American Society of Clinical Oncology, ASCO）大会上发布的最新结果发现，贝伐珠单抗联合治疗组的PFS显著优于单用厄洛替尼组（16.4个月*vs* 9.8个月；HR=0.52，95%CI：0.35- 0.76；*P*=0.000, 5）；且总体严重不良事件（serious adverse event, SAE）无显著组间差异，但贝伐珠单抗联合治疗组的3级及以上AE发生率显著高于厄洛替尼单药组（90.7% *vs* 53.2%），这主要归因于≥3级高血压事件。但绝大部分的高血压事件可通过抗高血压治疗得到控制^[34]^。

2019年4月发表于《*Lancet Oncology*》的Ⅲ期随机对照临床试验（NEJ026研究）旨在进一步确认JO25567研究的结果^[35]^。研究纳入228例未经治疗的*EGFR*突变阳性的晚期非鳞NSCLC患者，包括无症状脑转移患者。中位12.4个月随访的分析数据显示，厄洛替尼联合贝伐珠单抗组的中位PFS相比单用厄洛替尼组明显延长（16.9个月*vs* 13.3个月，HR=0.605，*P*=0.016）。亚组分析发现，19外显子缺失患者的PFS为（16.6个月*vs* 12.4个月；HR=0.69；95%CI：0.41-1.16），L858R突变患者PFS为（17.4个月*vs* 13.7个月；HR=0.57；95%CI：0.33-0.97）。安全性方面，≥3级AE发生率在贝伐珠单抗联合治疗组和厄洛替尼单药组分别为88%与46%，联合治疗组最常见的AE是皮疹（88% *vs* 87%），其次分别为腹泻（47% *vs* 41%）、高血压（46% *vs* 10%）、蛋白尿（32% *vs* 5%）、转氨酶升高（27% *vs* 30%）和出血（除外肺动脉高压；23% *vs* 3%）。研究期间未观察到治疗相关的死亡事件。

**推荐意见2**

**有*EGFR*敏感型突变的晚期非鳞NSCLC患者（含无症状脑转移患者）中，一线可选择使用厄洛替尼联合贝伐珠单抗[2A]。**

**重组人血管内皮抑制素用于晚期NSCLC一线治疗的推荐意见**

我国开展的一项随机、双盲、对照、多中心临床研究纳入了493例初治或经治的Ⅲ期/Ⅳ期NSCLC患者，随机分配给予长春瑞滨和顺铂（NP）联合重组人血管内皮抑制素（YH-16）（试验组）与NP联合安慰剂（对照组）治疗。在486例可评价疗效的患者中，试验组和对照组的ORR、临床获益率（clinical benefit rate, CBR）、中位TTP分别为35.4% *vs* 19.5%（*P*=0.000, 3）、73.3% *vs* 64.0%（*P*=0.035）、6.3个月*vs* 3.6个月（*P*=0.000）。在初治患者中，两组患者ORR、CBR、中位TTP分别为40.0% *vs* 23.9%（*P*=0.003）、76.5% *vs* 65.0%（*P*=0.023）、6.6个月*vs* 3.7个月（*P*=0.000）；在经治患者中，两个治疗组的ORR、CBR、中位TTP分别为23.9% *vs* 8.5%（*P*=0.034）、65.2% *vs* 61.7%（*P*=0.68）、5.7个月*vs* 3.2个月（*P*=0.000, 2）。此外，试验组的临床症状缓解率较对照组略高，但无统计学差异（*P* > 0.05），但试验组在治疗后QoL评分有明显提高（*P*=0.015, 5）。该研究^[36]^表明，相比单用NP方案，YH-16联合NP方案能明显改善初治以及经治的晚期NSCLC患者的治疗结局，且安全性良好。

**推荐意见3**

**对于驱动基因突变阴性，PS 0分-1分的晚期NSCLC患者（包括鳞状NSCLC和非鳞NSCLC），可一线使用重组人血管内皮抑制素联合长春瑞滨和顺铂治疗2个-4个周期[2B]，在可耐受的情况下，可适当延长重组人血管内皮抑制素使用时间[3类]。**

**抗血管生成药物联合肿瘤免疫治疗将成为未来NSCLC一线治疗的新方向**

大量临床研究证据证实，以程序性死亡因子-1（programmed death-1, PD-1）/程序性死亡因子配体-1（programmed death-ligand 1, PD-L1）单克隆抗体为代表的肿瘤免疫治疗在晚期NSCLC患者中有可靠的治疗效果。并且联合其他治疗药物，如抗血管生成药物，可扩大肿瘤免疫治疗的受益人群^[37]^。IMpower150研究是一项多中心、开放标签、随机、对照Ⅲ期临床研究，纳入1, 202例受试者，随机分配后分别接受Atezolizumab+卡铂+紫杉醇（ACP方案）、贝伐珠单抗+卡铂+紫杉醇（BCP方案）、Atezolizumab+贝伐珠单抗+卡铂+紫杉醇（ABCP方案），治疗4个-6个疗程（21 d为1个疗程）后，接受Atezolizumab或贝伐珠单抗或两者联合进行维持治疗。已公布的数据显示，在1, 040例（86.5%）*EGFR*/*ALK*突变阴性患者中，试验组ABCP组（356例）的中位PFS较对照组BCP组（336例）显著改善（8.3个月*vs* 6.8个月；HR=0.62；95%CI：0.52-0.74；*P* < 0.001）；在效应T细胞（Teff）高表达的野生型基因患者中观察到的结果与之一致（11.3个月*vs* 6.8个月，HR=0.51；95%CI：0.38-0.68；*P* < 0.001）。此外，在*EGFR*或*ALK*突变阳性患者（中位PFS结果：ABCP组9.7个月*vs* BCP组6.1个月，HR=0.59，95%CI：0.37-0.94）和肝转移患者（中位PFS结果：ABCP组7.4个月*vs* BCP组4.9个月，HR=0.59，95%CI：0.26-0.66）中同样观察到ABCP治疗较BCP方案可达更大PFS获益，在其他亚组人群，包括PD-L1低表达/不表达、Teff低表达、*KRAS*突变阳性患者中也同样观察到类似结果。研究中未发现新的安全性问题^[18]^。2019年3月更新的IMpower150研究*EGFR*突变和基线肝转移亚组结果显示，在敏感性*EGFR*突变亚组中，ABCP组较BCP组的OS显著改善[NE（95%CI：NE-NE）*vs* 17.5个月（95%CI: 11.7-NE）；HR=0.31（95%CI: 0.11-0.83）]；在基线伴肝转移的亚组患者中，ABCP组较BCP组的OS同样显著改善[13.3个月（11.6-NE）*vs* 9.4个月（7.9-11.7）；HR=0.52（0.33-0.82）]。但在总体人群及上述亚组中，ACP与BCP组的OS均无显著差异^[38]^。IMpower150研究首次证实ABCP方案治疗*EGFR*突变和基线肝转移患者获得良好的疗效。IMpower130研究旨在评估ACP *vs* CP方案一线治疗非鳞NSCLC的疗效和安全性，结果显示，与CP方案比较，ACP方案仅可改善总体人群[18.1个月（95%CI：15.3-20.8）*vs* 13.9个月（12.0-18.2）；HR=0.80（95%CI：0.65-0.99）；*P*=0.039]和*ALK*或*EGFR*野生型亚组[18.6个月（95%CI：16.0-21.2）*vs* 13.9个月（12.0-18.7）；HR=0.79（95%CI：0.64-0.98）；*P*=0.033]的OS，进一步对野生型患者进行亚组分析显示，ACP *vs* CP治疗肝转移患者的OS无显著差异[HR 1.04（0.63-1.72）]^[39]^。对比IMpower150和IMpower130研究结果发现，CP方案联合Atezolizumab方案在EGFR野生型和无肝转移患者中获益更高，对于*EGFR*突变和伴肝转移的患者，此三药联合获益不大。然而，在三药基础上加上贝伐珠单抗的ABCP方案却对*EGFR*突变和伴肝转移的患者获益更高，说明抗血管生成药物联合肿瘤免疫治疗具有增效作用，具有很大的临床应用潜力。因此专家组认为对于Ⅳ期或复发转移性非鳞NSCLC患者，Atezolizumab联合贝伐珠单抗应该是未来抗血管联合免疫治疗的一种重要新策略，特别是敏感性*EGFR*突变和肝转移患者。

**一线治疗后的维持治疗**

在一线治疗结束后，单用贝伐珠单抗维持治疗的疗效得到了大样本研究数据的支持。2011年，一项研究回顾性分析了美国癌症网络的电子病历资料，结果显示，晚期非鳞NSCLC患者接受一线标准化疗联合贝伐珠单抗治疗后，继续使用贝伐珠单抗单药维持，患者的中位OS与PFS分别为20.9个月与10.3个月，明显优于未接受维持治疗患者的10.2个月与6.5个月（*P*均 < 0.001）^[40]^。ARIES研究纳入1, 967例接受标准一线化疗的晚期非鳞NSCLC患者，结果显示，一线化疗后使用贝伐珠单抗维持治疗患者在诱导治疗后的中位OS明显优于未接受维持治疗患者（15.6个月*vs* 11.3个月，*P* < 0.001）^[41]^。

2013年发表的AVAPERL研究在晚期非鳞NSCLC患者分析了一线培美曲塞/顺铂联合贝伐珠单抗治疗后使用贝伐珠单抗单药维持或培美曲塞/贝伐珠单抗联合方案维持治疗的疗效，最终证明联合方案维持治疗组患者的PFS和OS均长于单药维持组[7.4个月*vs* 3.7个月（HR=0.48；95%CI：0.35-0.66；*P* < 0.001）]。联合方案维持治疗组的所有级别、≥3级，以及严重不良事件更为常见，但未观察到新的安全性事件^[25]^。

PointBreak Ⅲ期研究观察到了类似的结果^[24]^。该研究纳入939例未经治疗的Ⅲb期或Ⅳ期非鳞NSCLC患者，随机分配给予一线贝伐珠单抗+卡铂+培美曲塞（PemCBev组）或贝伐珠单抗+卡铂+紫杉醇（PacCBev组）治疗，如一线治疗后无进展或达到稳定，PemCBev组给予贝伐珠单抗+培美曲塞维持治疗，PacCBev组给予贝伐珠单抗维持治疗，维持治疗持续至肿瘤进展。结果显示，PemCBev组的292例受试者和PacCBev组的298例受试者接受了维持治疗。PemCBev组维持治疗患者的中位OS达到17.7个月，PacCBev组达到15.7个月。PemCBev组维持治疗患者的中位PFS达到8.6个月，PacCBev组达到6.9个月。研究中未观察到新的安全性问题。

2019年ASCO期间发表的COMPASS研究纳入既往未接受过治疗的Ⅲb期/Ⅳ期、*EGFR*野生型非鳞NSCLC患者，接受卡铂+培美曲塞+贝伐珠单抗诱导治疗后，根据维持治疗方案随机分为贝伐珠单抗单药维持组和贝伐珠单抗+培美曲塞维持治疗组。结果显示，与贝伐珠单抗组比较，贝伐珠单抗+培美曲塞组的中位OS呈延长趋势（19.6个月*vs* 23.3个月；HR=0.87；95%CI：0.73-1.05，*P*=0.069），PFS显著延长（4.0个月*vs* 5.7个月；HR=0.67；95%CI：0.57-0.79，*P* < 0.001）^[42]^。

**推荐意见4**

**对于驱动基因突变阴性，PS 0分-1分的晚期非鳞NSCLC患者，贝伐珠单抗联合含铂双药一线治疗后达到缓解或疾病稳定，推荐使用贝伐珠单抗单药维持治疗，直至患者不可耐受或出现疾病进展[1A]。如患者一线使用贝伐珠单抗+铂类+培美曲塞方案，可选择贝伐珠单抗联合培美曲塞维持治疗直至患者不可耐受或出现疾病进展[2A]。**

**二线治疗**

2016年ASCO大会上发布了一项随机对照Ⅲ期临床研究（IFCT-1103 ULTIMATE）结果，在166例接受过含铂化疗的晚期非鳞NSCLC患者中比较了贝伐珠单抗联合多西他赛和单用多西他赛的疗效^[43]^。随访28.9个月的结果显示，贝伐珠单抗联合多西他赛作为二线或三线治疗，相比单用多西他赛可明显改善中位PFS（5.4个月*vs* 3.9个月，HR=0.56，*P*=0.01）。与单用多西他赛比较，贝伐珠单抗联合多西他赛治疗组的ORR也明显提高（22.5% *vs* 5.5%, *P*=0.006）。

2017年发表的一项回顾性研究进一步支持上述研究结果^[44]^。该研究比较了在实际临床中接受贝伐珠单抗联合多西他赛（*n*=15）和单用多西他赛（*n*=55）作为二线及以上治疗患者的临床结局。结果显示，贝伐珠单抗联合多西他赛组患者的ORR（26.7% *vs* 9.1%）和PFS（5.9个月*vs* 2.1个月，*P*=0.081）均相对单用多西他赛组患者呈改善趋势。

**推荐意见5**

**既往化疗失败，且未使用过贝伐珠单抗治疗的晚期非鳞NSCLC患者，可选择贝伐珠单抗联合多西他赛用于二线及以上治疗[2A]。**

**三线及以上治疗**

一项安慰剂对照、随机、双盲、多中心Ⅱ期临床试验（ALTER0302研究）旨在明确安罗替尼用于复发性晚期NSCLC三线及以上的治疗效果和安全性。研究纳入117例至少接受过二线方案治疗的复发性Ⅲb期/Ⅳ期肺鳞癌或肺腺癌患者，PS 0分-2分，随机分配给予安罗替尼或安慰剂治疗。结果显示，安罗替尼组患者（*n*=60）的PFS（4.8个月*vs* 1.2个月，HR=0.32，*P* < 0.000, 1）与ORR（10.0% *vs* 0%, *P*=0.028）均显著优于安慰剂组（*n*=57），但两组的中位OS无显著差异（9.3个月*vs* 6.3个月；HR=0.78，*P*=0.231, 6）^[21]^。

后续开展的Ⅲ期临床试验（ALTER0303研究）共437例患者接受随机分配并完成了研究治疗。总体人群数据显示，相比安慰剂组，安罗替尼组（*n*=294）的中位OS延长3.3个月（9.6个月*vs* 6.3个月，HR=0.68，*P*=0.001, 8）；中位PFS延长4.0个月（5.4个月*vs* 1.4个月，HR=0.25，*P* < 0.000, 1）。安罗替尼组的ORR（9.2% *vs* 0.7%, *P* < 0.000, 1）和DCR（81.0% *vs* 37.1%, *P* < 0.000, 1）等次要终点也均显著优于对照组。安罗替尼在研究中最常见的≥3级不良反应是高血压和低血钠症^[22]^。

**推荐意见6**

**对于驱动基因突变阴性以及*EGFR*突变阳性的复发性晚期NSCLC（包括鳞癌和非鳞癌）患者，推荐安罗替尼作为三线及以上治疗。对于存在*EGFR*基因突变或*ALK*突变阳性的患者，应在接受相应的靶向药物治疗后进展且至少接受过2种系统化疗后出现进展或复发后使用安罗替尼[1A]。**

**特定人群的治疗推荐**

**老年晚期非鳞NSCLC患者**

Ⅳ期MO19390（SAiL）研究在623例晚期非鳞患者中进行的年龄亚组分析显示，年龄较高（≥65岁）和较低（< 65岁）患者一线接受贝伐珠单抗联合任意标准化疗方案（含铂或不含铂）治疗的结局无明显差异：中位OS均为14.6个月，中位TTP分别为8.2个月与7.6个月，缓解率分别为49.3%和52.4%，疾病控制率分别为89.3%和88.4%。而且两组出血、高血压、蛋白尿不良反应发生率亦无显著差异^[45]^。

ARIES研究的年龄亚组分析结果则显示，一线接受含贝伐珠单抗方案治疗后，≥65岁或者≥75岁患者的中位PFS与 < 65岁或者 < 75岁的患者类似，分别为6.4个月*vs* 6.8个月与6.6个月*vs* 6.6个月；但OS则短于较年轻患者，分别为14.2个月*vs* 12.1个月与13.5个月*vs* 11.6个月。老龄（≥70岁）患者接受含贝伐珠单抗一线治疗后动脉血栓发生率为3%，而较年轻患者则为2%，其他不良反应发生率与总体人群相似^[46]^。

**推荐意见7**

**对于晚期老年非鳞NSCLC患者，一线治疗可选择含贝伐珠单抗方案[2A]。**

ALTER 0303 Ⅲ期研究中包括了28例老年患者，老年患者亚组结果显示，安罗替尼治疗患者的OS（14.5个月*vs* 6.3个月，HR=0.34，*P*=0.03）较安慰剂组明显改善。ALTER 0303研究的亚组分析表明，安罗替尼可作为晚期NSCLC老年患者的三线治疗选择^[22]^。

**推荐意见8**

**对于晚期老年NSCLC患者（年龄 > 70岁），三线治疗可选择安罗替尼[2A]。**

**伴脑转移的晚期非鳞NSCLC患者**

一项纳入17项研究的回顾性探索性分析显示，脑转移肿瘤患者（包括乳腺癌、NSCLC、肾癌、结直肠癌）接受贝伐珠单抗治疗后的颅内出血发病率为0.8%-3.3%，且与对照组无显著差异，这表明贝伐珠单抗并未增加脑转移患者的颅内出血风险^[47]^。另一项针对伴脑转移NSCLC患者的系统文献综述^[48]^也发现，未治或经治的NSCLC脑转移患者接受贝伐珠单抗等抗VEGF靶向药物治疗后，颅内出血风险未显著增加。

一项前瞻性非对照Ⅱ期临床试验（BRAIN研究）在91例无症状脑转移非鳞NSCLC患者中分析了贝伐珠单抗治疗的效果及安全性。67例患者接受贝伐珠单抗联合卡铂+紫杉醇一线治疗。结果显示，贝伐珠单抗方案一线治疗患者的中位PFS为6.7个月（95%CI: 5.7-7.1），中位OS为16.0个月，ORR为62.7%。一线治疗相关不良反应均可控^[49]^。

**推荐意见9**

**对伴脑转移的晚期非鳞NSCLC患者，贝伐珠单抗联合化疗的疗效和安全性良好，可作为一线选择方案[1A]。**

**伴恶性胸腔积液的晚期非鳞NSCLC患者**

一项中国研究纳入72例伴发恶性胸腔积液的晚期转移性非鳞NSCLC患者，分析局部胸腔灌注贝伐珠单抗联合顺铂的治疗效果。结果显示，联合治疗组患者的胸腔积液控制率显著高于顺铂单药治疗组（88.33% *vs* 50.00%, *P* < 0.05）；胸水中的VEGF水平在治疗后也明显降低（*P* < 0.01），降低幅度明显大于单用顺铂组（*P* < 0.01）；此外，在VEGF高表达患者中，贝伐珠单抗联合化疗局部灌注治疗的治疗有效率更高（*P* < 0.001）。治疗过程中患者的耐受性良好^[50]^。

2017年，一项中国前瞻性、随机对照、多中心Ⅲ期临床研究纳入317例伴有中等量以上恶性胸腹腔积液的患者，旨在观察和证实腔内应用重组人血管内皮抑制素注射液（恩度）和/或顺铂治疗恶性胸腹腔积液的有效性和安全性。结果显示，恩度单药组（A组）、顺铂单药组（B组）和恩度联合顺铂组（C组）的ORR分别为48.51%、46.39%和63.00%（*P*=0.037, 3），两两比较，C组较A组、B组高（*P*=0.018, 9）；对于血性胸腔积液，A组、C组ORR分别为71.42%和88.88%，均显著优于B组的40.00%（*P*=0.001, 3）。A组、B组、C组的中位TTP分别为68.869 d、44.951 d和69.030 d（*P*=0.012, 1），两两比较，A组、C组的中位TTP均长于B组（*P*=0.024, 0, *P*=0.004, 6）。安全性方面，A组不良事件发生率显著低于B组（*P*=0.000, 5），B组、C组之间差异无统计学意义（*P*=0.286, 6）^[51]^。

**推荐意见10**

**伴有恶性胸腔积液的晚期非鳞NSCLC患者，可在全身治疗的基础上局部使用贝伐珠单抗或重组人血管内皮抑制素[2B]。**

**不良反应管理**

从临床研究的数据来看，抗血管生成药物用于晚期NSCLC患者治疗期间的不良反应可控，安全性良好。常见的3级及以上不良事件包括高血压、蛋白尿、出血和血栓栓塞事件等。值得一提的是，贝伐珠单抗目前已在全球获批7个肿瘤适应证，已有270万人群在实际临床中使用过这一药物，临床对其安全性的预控性更高。

**NSCLC抗血管生成药物临床应用注意事项**

·了解用药风险因素，规范使用抗血管生成药物是降低不良反应发生风险的重要前提，对于某些特殊人群需要用到抗血管生成药物时建议慎重评估患者风险，必要时请研究者在专科医生参与指导下用药。

·以下因素可能增加出血/咯血风险，使用抗血管生成药物时应慎重：伴有空洞或者中央型鳞状细胞NSCLC、长期或大剂量使用抗风湿/抗炎药物治疗或抗凝治疗的患者、原发病灶比较大且该病灶接受过放射治疗的患者、既往具有动脉硬化症病史的患者、具有消化性溃疡的患者等。

· 3个月内发生过肺出血/咯血（> 1/2茶匙的鲜红血液）的患者不应该使用抗血管生成药物治疗。

·有动脉血栓栓塞史，房颤、血管支架植入术后或糖尿病的患者，在抗血管治疗过程中发生动脉血栓栓塞的风险增高。在采用抗血管生成药物对此类患者进行治疗时，应该慎重。

·有临床重度心血管病的患者（如有冠心病史或充血性心力衰竭），使用抗血管生成药物时应谨慎。

·重大手术后至少28 d之内不应该开始抗血管生成药物治疗，或者应该等到手术伤口完全愈合之后再开始。抗血管治疗过程中发生了伤口不愈合等并发症的患者，应该暂停抗血管生成药物治疗，直到伤口完全愈合。需要进行择期手术的患者也应该暂停抗血管生成药物治疗。

·贝伐珠单抗可能损害女性生育力。因此，在使用贝伐珠单抗治疗前，应当与有潜在生育力的妇女讨论生育力的保护方法。妊娠期间不应该使用贝伐珠单抗。育龄妇女在采用贝伐珠单抗进行治疗时，应采取适当的避孕措施。

·建议妇女在采用贝伐珠单抗进行治疗时停止哺乳，并且在最后一次贝伐珠单抗治疗后的至少6个月内不要采取母乳喂养。

**抗血管生成药物治疗相关不良事件的临床管理策略**

见表 2。

**2:** 不良事件的临床管理策略 Clinical management strategies for adverse events

高血压	
≥3级发生率	贝伐珠单抗：5%-9%，安罗替尼：10.0%-13.6%
发生机制	①血管内皮生长因子（vascular endothelial growth factor, VEGF）被阻断，导致一氧化氮水平下降，进而导致血管无法扩张，增加外周阻力，引发高血压； ②一氧化氮水平较低还与肾排泄量减少相关，继而导致水钠储溜^[52]^。
推荐意见11	·使用贝伐珠单抗或安罗替尼治疗时，需动态监测患者血压值； ·如发生高血压，或患者血压值较基线明显升高，则推荐开始使用降压药，达到良好的血压控制，推荐低危患者的血压控制目标是140 mmHg/90 mmHg，高危患者应为130 mmHg/80 mmHg； ·血管紧张素转化酶抑制剂（angiotensin-converting enzyme inhibitor, ACEI）、血管紧张素Ⅱ受体拮抗剂（angiotensin Ⅱ receptor antagonist, ARB）、*β*受体阻滞剂、钙离子通道阻滞剂都是可选择的降压药物； ·如出现中度以上的高血压（高于160 mmHg/100 mmHg），且降压药暂不能控制，则应暂停贝伐珠单抗或安罗替尼，直至血压恢复至可控状态，如果高血压经过治疗1个月仍未得到控制或出现高血压危象或高血压性脑病，则需停用贝伐珠单抗或安罗替尼。
蛋白尿	
≥3级发生率	贝伐珠单抗： < 1%-4%，安罗替尼：0%-2.4%
发生机制	抑制VEGF通路则会导致内皮失窗孔化、内皮水肿和脱离，进而破坏滤过屏障的完整性，导致蛋白尿^[53]^。
推荐意见12	·在每次开始贝伐珠单抗或安罗替尼治疗前都应进行尿蛋白的检测； ·如果出现24 h蛋白尿水平 > 2 g，应该暂停贝伐珠单抗或安罗替尼治疗，并密切观察，直至24 h蛋白尿水平 < 2 g； ·肾病综合征（24 h蛋白尿水平 > 3.5 g）患者建议停用贝伐珠单抗或安罗替尼； · ACEI和ARB类降压药可降低蛋白尿的严重程度和终末期肾病的风险，推荐使用。
出血	
≥3级发生率	贝伐珠单抗：出血发生率为1.0%-4.4%；安罗替尼：咯血发生率为0%-3.1%
发生机制	① VEGF失活可能会影响血小板的活化； ②阻断VEGF通路会影响内皮细胞存活和增殖，导致血管完整性受损，特别是在具有高VEGF依赖性的组织中，如气道黏膜受损； ③阻断VEGF导致一氧化氮水平下调也可能会影响血小板的活化^[53]^。
推荐意见13	·在开始治疗前应评价潜在风险因素，鉴别出血高风险人群，如：存在活动性胃溃疡会增加胃肠道出血风险；空洞型肺鳞癌患者存在肺出血风险； ·近期瘤块中有出血征象的患者，使用抗血管生成药物时应持谨慎态度； · 3个月内发生过肺出血/咯血（> 1/2茶匙的鲜红血液）的患者不应该使用贝伐珠单抗或者安罗替尼治疗； ·监测患者的中枢神经系统出血症状和体征，一旦出现颅内出血应该中断贝伐珠单抗或者安罗替尼治疗； ·治疗过程中发生1级出血事件，不需调整抗血管生成药物剂量；发生2级出血事件，需暂停治疗；发生≥3级出血事件，应该永久停用贝伐珠单抗或安罗替尼。
血栓栓塞事件	
≥3级发生率	贝伐珠单抗：0%-7%
发生机制	①阻断VEGF可能影响受损血管内皮表面的修复过程，导致内皮组织暴露以及内皮细胞的凋亡，进而引发凝血级联反应，形成血凝块；细胞凋亡可能导致磷酯酰丝氨酸的重新分布，后者可增强凝血酶原因子X的促凝活性；在凋亡细胞中还会出现抗凝血因子血栓调节蛋白和硫酸乙酰肝素的下调； ②阻断VEGF还可以刺激凝血酶原因子Ⅲ，触发凝血过程^[53]^。
推荐意见14	·对使用抗血管生成药物治疗中出现静脉血栓栓塞事件（venous thromboembolism, VTE）的晚期NSCLC患者，应停止治疗，并推荐使用低分子量肝素（low molecular weight heparin, LMWH）进行抗凝治疗； ·对于出现≤3级VTE的患者，在开始LMWH后可恢复抗血管生成药物治疗；对于出现≥4级VTE或抗凝治疗后复发性或难治性血栓栓塞的患者，应终止抗血管生成药物治疗； ·所有使用贝伐珠单抗的患者都应考虑存在动脉血栓栓塞事件（arterial thromboembolism，ATE）风险，有动脉血栓栓塞史、糖尿病或年龄 > 65岁以及易发血管病（如心脏支架植入史）的患者，使用贝伐珠单抗时应慎重； ·治疗过程中出现任何级别的ATE事件，急性期应中止贝伐珠单抗治疗； ·近期发生过ATE的患者，至少在ATE发生后6个月内不能使用贝伐珠单抗治疗，开始贝伐珠单抗治疗前应确定患者处于稳定状态或无症状。必要时请专科医生会诊。

## 5 抗血管生成药物在晚期NSCLC患者中的临床应用总结

见表 3。具体实践可参考附件：抗血管生成药物临床不良反应处理手册（http://dx.doi.org/10.3779/j.issn.1009-3419.2019.07.11）。

**3:** 推荐意见汇总 Summary of recommendations

一线治疗
·驱动基因突变阴性、PS 0分-1分晚期非鳞NSCLC患者： 贝伐珠单抗联合含铂双药方案作为一线治疗选择[1A]。 ·驱动基因突变阴性、PS 0分-1分晚期NSCLC患者（包括鳞癌和非鳞癌）： 重组人血管内皮抑制素联合长春瑞滨和顺铂治疗2个-4个周期[2B]，在可耐受的情况下，可适当延长重组人血管内皮抑制素使用时间[3类]。 ·有*EGFR*敏感突变的晚期非鳞NSCLC患者（含无症状脑转移患者）： 厄洛替尼联合贝伐珠单抗[2A]。
一线维持治疗
·驱动基因突变阴性，PS 0分-1分晚期非鳞NSCLC患者，贝伐珠单抗联合含铂双药一线治疗后达到缓解或疾病稳定： 贝伐珠单抗单药维持直至不可耐受或出现疾病进展[1A]。 如患者一线使用贝伐珠单抗+铂类+培美曲塞方案，可选择贝伐珠单抗联合培美曲塞维持治疗直至患者不可耐受或出现疾病进展[2A]。
二线治疗
·既往治疗未使用过贝伐珠单抗治疗晚期非鳞NSCLC患者： 贝伐珠单抗联合多西他赛用于二线及以上治疗[2A]。
三线及以上治疗
·驱动基因突变阴性的复发性晚期NSCLC（包括鳞癌和非鳞癌）患者： 安罗替尼单药[1A]。 ·存在*EGFR*基因突变或*ALK*突变阳性的患者： 应在接受相应的靶向药物治疗后进展且至少接受过两种系统化疗后出现进展或复发后使用安罗替尼[1A]。
特定人群的治疗
伴恶性胸腔积液的晚期NSCLC ·伴有恶性胸腔积液的晚期非鳞NSCLC患者： 可在全身治疗的基础上局部使用贝伐珠单抗或重组人血管内皮抑制素[2B]。 伴脑转移的晚期NSCLC ·伴脑转移的晚期非鳞NSCLC患者： 贝伐珠单抗联合化疗可作为一线选择方案[2A]。 老年患者 ·晚期非鳞NSCLC老年患者： 一线治疗可选择含贝伐珠单抗方案[1A]。 · > 70岁的晚期NSCLC高龄患者： 三线治疗可选择安罗替尼[2A]。
不良反应管理
·高血压 ➢使用贝伐珠单抗或安罗替尼治疗时，需动态监测患者血压值； ➢如发生高血压，或患者血压值基线明显升高，则推荐开始使用降压药，达到良好的血压控制，推荐低危患者的血压控制目标是140 mmHg/90 mmHg，高危患者应为130 mmHg/80 mmHg； ➢血管紧张素转化酶抑制剂（angiotensin-converting enzyme inhibitor, ACEI）、血管紧张素Ⅱ受体拮抗剂（angiotensin Ⅱ receptor antagonist, ARB）、*β*受体阻滞剂、钙离子通道阻滞剂都是可选择的降压药物； ➢如出现中度以上的高血压（高于160 mmHg/100 mmHg），则应暂停贝伐珠单抗或安罗替尼，直至血压恢复至可控状态，如果高血压经过治疗1个月仍未得到控制或出现高血压危象或高血压性脑病，则需停用贝伐珠单抗或安罗替尼。
·蛋白尿 ➢在每次开始贝伐珠单抗或安罗替尼治疗前都应进行尿蛋白的检测； ➢如果出现24 h蛋白尿水平 > 2 g，应该暂停贝伐珠单抗或安罗替尼治疗，并密切观察，直至24 h蛋白尿水平 < 2 g； ➢肾病综合征（24 h蛋白尿水平 > 3.5 g）患者建议停用贝伐珠单抗或安罗替尼； ➢ ACEI和ARB类降压药可降低蛋白尿的严重程度和终末期肾病的风险，推荐使用。
·出血 ➢在开始治疗前应评价潜在风险因素，鉴别出血高风险人群，如：存在活动性胃溃疡会增加胃肠道出血风险；空洞型肺鳞癌患者存在肺出血高风险； ➢近期瘤块中有出血征象的患者，使用抗血管生成药物时应持谨慎态度； ➢ 3个月内发生过肺出血/咯血（> 1/2茶匙的鲜红血液）的患者不应该使用贝伐珠单抗或者安罗替尼治疗； ➢监测患者的中枢神经系统出血症状和体征，一旦出现颅内出血应该中断贝伐珠单抗或者安罗替尼治疗； ➢治疗过程中发生1级出血事件，不需调整抗血管生成药物剂量；发生2级出血事件，需暂停治疗；发生≥3级出血事件，应该永久停用贝伐珠单抗或安罗替尼。
·血栓栓塞 ➢对使用抗血管生成药物治疗中出现静脉血栓栓塞事件（venous thromboembolism, VTE）的晚期NSCLC患者，应停止治疗，并推荐使用低分子量肝素（low molecular weight heparin, LMWH）进行抗凝治疗； ➢对于出现≤3级VTE的患者，在开始LMWH后可恢复抗血管生成药物治疗；对于出现≥4级VTE或抗凝治疗后复发性或难治性血栓栓塞的患者，应终止抗血管生成药物治疗； ➢所有使用贝伐珠单抗的患者都应考虑存在动脉血栓栓塞事件（arterial thromboembolism，ATE）风险，有动脉血栓栓塞史、糖尿病或年龄 > 65岁以及易发血管病（如心脏支架植入史）的患者，使用贝伐珠单抗时应慎重； ➢治疗过程中出现任何级别的ATE事件，急性期应中止贝伐珠单抗治疗； ➢近期发生过ATE的患者，至少在ATE发生后6个月内不能使用贝伐珠单抗治疗，开始贝伐珠单抗治疗前应确定患者处于稳定状态或无症状。

## 6 总结与展望

从传统的含铂双药化疗到近10年间得以应用的分子靶向治疗以及近四年来逐渐兴起的肿瘤免疫治疗，晚期NSCLC的治疗理念在不断地发生变化，而自美国食品药品监督管理局（Food and Drug Administration, FDA）于2006年批准抗血管生成药物贝伐珠单抗用于晚期NSCLC一线治疗以来，从大量的临床数据和实际临床应用情况来看，这类药物在晚期NSCLC患者的临床管理中一直占据着重要地位。肿瘤新生血管生成与包括NSCLC在内的实体瘤的发生和进展密切相关，且贯穿肿瘤进展的全程，因此抗血管生成药物可使广泛的晚期NSCLC患者达到治疗获益。此外，抗血管生成药物因其特有的抗血管通透机制，可在恶性胸腔积液和脑水肿患者中发挥治疗作用。相比欧美人群，东亚患者可从抗血管生成药物治疗中得到更大获益。随着3个抗血管生成药物在我国陆续获批用于晚期NSCLC患者，这类药物在我国肺癌临床已实际应用了近十年，积累了丰富的经验和真实世界数据。贝伐珠单抗已在全球临床应用十余年，获批了7个适应证，已有270万人实际使用过该药，该药在我国的获批是基于大样本的中国人群数据。重组人血管内皮抑制素和安罗替尼则是我国自主研发的抗血管生成药物。这3个药物在中国晚期NSCLC患者中的疗效和安全性均得到了强有力的证据支持。对于抗血管生成药物在晚期NSCLC患者中的应用，还有许多问题有待进一步的研究探讨。首先，探讨最佳的联合治疗新方案是下一步研究的重要方向，从目前的研究结果来看，抗血管生成药物联合新型靶向药物以及新兴的肿瘤免疫治疗都可能成为将来临床治疗晚期NSCLC的重要策略。第二，抗血管生成药物在晚期NSCLC患者中的最佳给药模式还有待明确；第三，确定疗效生物标志物是选择优势人群和实现精准抗血管生成治疗的前提。但对于抗血管生成药物在NSCLC中治疗有效性的相关生物标志物的探索，目前仍处于初始阶段。

**Table:** 晚期非小细胞肺癌抗血管生成药物治疗专家组成员（按姓氏汉语拼音排名）

	医院	姓名
组长	上海交通大学附属胸科医院	韩宝惠
组长	天津医科大学肿瘤医院	李凯
组长	同济大学附属上海市肺科医院	周彩存
顾问组长	吉林省肿瘤医院	程颖
顾问组长	上海东方医院	李进
顾问组长	四川大学华西医院	卢铀
顾问组长	中国人民解放军第八一医院	秦叔逵
顾问组长	天津医科大学肿瘤医院	王长利
顾问组长	中国医学科学院肿瘤医院	王洁
顾问组长	山东省肿瘤医院	王哲海
顾问组长	中山大学附属肿瘤医院	张力
顾问组长	四川大学华西医院	周清华
执笔人	上海交通大学附属胸科医院	储天晴
参与专家	安徽省立医院	操乐杰
参与专家	复旦大学附属肿瘤医院	常建华
参与专家	湖南省肿瘤医院	陈建华
参与专家	浙江省肿瘤医院	陈明
参与专家	浙江大学附属第一医院	周建英
参与专家	浙江省肿瘤医院	范云
参与专家	北京大学肿瘤医院	方健
参与专家	上海交通大学附属胸科医院	傅小龙
参与专家	中国人民解放军第三零七医院	高红军
参与专家	中国医学科学院肿瘤医院	高树庚
参与专家	福建省肿瘤医院	黄诚
参与专家	中国人民解放军总医院	焦顺昌
参与专家	南京鼓楼医院	刘宝瑞
参与专家	浙江大学医学院附属邵逸夫医院	楼海舟
参与专家	青岛市中心医院	马学真
参与专家	浙江大学医学院附属邵逸夫医院	潘宏铭
参与专家	江苏省肿瘤医院	史美祺
参与专家	东部战区总医院	宋勇
参与专家	同济大学附属上海市肺科医院	苏春霞
参与专家	江苏省人民医院	束永前
参与专家	天津市肿瘤医院	王晶
参与专家	河南省肿瘤医院	王启鸣
参与专家	哈尔滨医科大学附属第二医院	徐玉清
参与专家	青岛大学附属医院	于壮
参与专家	上海瑞金医院	张俊
参与专家	青岛大学附属医院	张晓春
参与专家	浙江大学附属第一医院	赵琼
参与专家	复旦大学附属肿瘤医院	赵伟新
参与专家	上海新华医院	郑磊贞
参与专家	上海交通大学附属胸科医院	钟华
学术秘书	同济大学附属上海市肺科医院	何雅億
学术秘书	上海交通大学附属胸科医院	余科科
学术秘书	天津医科大学肿瘤医院	张翠翠
